# The Seed Coat’s Impact on Crop Performance in Pea (*Pisum sativum* L.)

**DOI:** 10.3390/plants11152056

**Published:** 2022-08-06

**Authors:** Teagen D. Quilichini, Peng Gao, Bianyun Yu, Dengjin Bing, Raju Datla, Pierre Fobert, Daoquan Xiang

**Affiliations:** 1 Aquatic and Crop Resource Development, National Research Council Canada, Saskatoon, SK S7N 0W9, Canada; 2 Saskatoon Research and Development Centre, Agriculture and Agri-Food Canada, 107 Science Place, Saskatoon, SK S7N 0X2, Canada; 3 Lacombe Research and Development Centre, Agriculture and Agri-Food Canada, 6000 C and E Trail, Lacombe, AB T4L 1W1, Canada; 4 Global Institute for Food Security, University of Saskatchewan, Saskatoon, SK S7N 4L8, Canada; 5 Sustainable Protein Production Program, National Research Council Canada, Ottawa, ON K1A 0R6, Canada

**Keywords:** seed development, embryogenesis, embryo, seed coat, legume, nutrition, *Pisum sativum* L.

## Abstract

Seed development in angiosperms produces three genetically and developmentally distinct sub-compartments: the embryo, endosperm, and seed coat. The maternally derived seed coat protects the embryo and interacts closely with the external environment especially during germination and seedling establishment. Seed coat is a key contributor to seed composition and an important determinant of nutritional value for humans and livestock. In this review, we examined pea crop productivity through the lens of the seed coat, its contribution to several valued nutritional traits of the pea crop, and its potential as a breeding target. Key discoveries made in advancing the knowledge base for sensing and transmission of external signals, the architecture and chemistry of the pea seed coat, and relevant insights from other important legumes were discussed. Furthermore, for selected seed coat traits, known mechanisms of genetic regulation and efforts to modulate these mechanisms to facilitate composition and productivity improvements in pea were discussed, alongside opportunities to support the continued development and improvement of this underutilized crop. This review describes the most important features of seed coat development in legumes and highlights the key roles played by the seed coat in pea seed development, with a focus on advances made in the genetic and molecular characterization of pea and other legumes and the potential of this key seed tissue for targeted improvement and crop optimization.

## 1. Introduction

Agricultural innovations to support growing demands placed on food production are urgently needed. As the world’s population continues to grow and demands for dietary protein and plant-based food rise amidst environmental challenges, the value of nutrient dense crop products is anticipated to increase. Legumes in the Fabaceae (or Leguminosae) represent the third largest Angiosperm family and contribute to over a quarter of the world’s crop productivity. Legumes offer exceptional nutritional value for human and animal diets, as reserves stored in embryonic cotyledons enable seeds to accumulate high levels of protein, starch, essential minerals, and vitamins. Further, the high protein content of legume seeds is commonly nitrogen (N)-fortified, as root symbiotic associations with bacteria and fungi provide most members of this family with N fixation capabilities [1]. N-containing essential amino acids complement cereal-based diet deficiencies, and the low-input farming and soil quality improvements provided by legume N fixation support sustainable agricultural practices [2,3]. Many of the key nutritional attributes of legume seeds are imparted or supported by the seed coat. The legume seed coat provides a multi-layered protective barrier, a conduit for maternal communication and nutrient allocation, and accumulates of a range of beneficial metabolites that are gaining recognition for their antioxidant and free radical scavenging activities [4]. Advances in genomic resources for legume crops offer great promise to support the targeted breeding and engineering of legumes, in order to efficiently target agronomic seed traits of interest for sustainable, nutritious legume crops that perform in the face of climate change.

**Figure 1:**
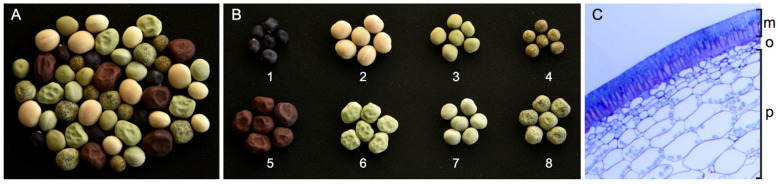
Seed coat diversity and structure in *Pisum sativum* L. (**A**) Selected dry pea seeds at maturity illustrate the breadth of seed coat color, size, and texture phenotypes that are attributed to the seeds. (**B**) Seed coat enriched in proanthocyanidins appear purple (1-22770A) and red (5-No. 3257). Seeds lacking proanthocyanidins appear yellow (2-CDC Amarillo). The color of the seed coat can be homogeneous (1-22770A, 3-PLP 174, 5-No. 3257, and 6-Kairyo Aotenashi), variegated (4-PLP 70 and 7-Uladovskij 208) or speckled (8-Biselia). (**C**) Cross-section of the pea seed (2-CDC Amarillo) revealing layers of a maturing seed coat. Layers visible here include an outermost epidermis of macrosclereids (m), a subepidemis of osteosclereids (o), and underlying seed coat parenchyma (p), with the chlorenchyma sub-layer visible.

*Pisum sativum* L. (pea) has played a prominent role in plant research since the 19th century, when Gregor Mendel’s observations of pea trait inheritance patterns initiated the field of genetics [5]. Seeds of green or yellow cotyledon pea varieties, also known as field, grain, and smooth or dry peas, constitute the primary source of peas grown for consumption [6]. In contrast to the immature peas produced by vegetable, fresh, or garden pea varieties, dry pea seeds are harvested following their maturation and natural desiccation. As a widely cultivated crop, selection and long-term breeding efforts have established pea varieties with a wide range of agronomically valued characters that impact the size, color, texture, nutritional composition, and digestibility of seeds (Figure 1A,B). The vast stockpiles of pea germplasm represent an immense potential for genetics-guided breeding and targeted genetic improvements and have provided a broad framework for understanding seed biology in legumes [5,7]. While pea cultivars on the market have facilitated dramatic yield and cultivation efficiency improvements, wild and ancestral pea varieties have been touted as valued sources for genetic variability and valuable traits [8,9]. In addition, a number of poorly characterized and regionally grown legumes including *Vigna unguiculata* (cowpea), *Phaseolus acutifolius* (tepary bean), and *Lathyrus sativus* (grass pea), known as orphan crops, produce nutrient dense seeds and growth habits under challenging growth conditions, as well as offering tremendous genetic diversity and the potential for trait discovery and introgression to improve mainstream legume production [3]. The rapid growth and widespread application of genomic technologies have produced a vast stockpile of large-scale plant genomic data for many legume species. The recent release of a pea reference genome suggests an exciting time for advancements in pea seed biology is in store, as in-depth mining and characterization of the sequenced genome, along with expression profiling and comparative analyses, enable functional characterization of key traits and expand understandings of molecular and physiological processes unique to pea seeds [10].

Here, we examined pea crop productivity through the lens of the seed coat, its contribution to the valued nutritional traits of the pea crop, and its potential as a breeding and engineering target. Key seed coat discoveries made in the sensing and transmission, architecture, and chemistry of the seed coat in pea and relevant legume relatives were discussed. Several seed coat traits, known mechanisms of genetic regulation, and efforts to modulate these mechanisms to facilitate composition and productivity improvements in pea were discussed, alongside opportunities to support the continued development of this nutritious but underutilized crop.

## 2. The Interconnected Sub-Compartments of the Pea Seed

The embryo, endosperm, and seed coat represent the three major sub-compartments of the seed and form a medley of filial and maternally derived tissues with highly coordinated developmental programs. In angiosperms, double-fertilization initiates seed formation from the ovary [11,12,13,14], with the fusion of sperm nuclei with the egg and central cells triggering embryo and endosperm development, respectively [15]. The triploid endosperm that fills the early embryonic sac serves a transitory role in embryonic nourishment and is absent from the mature seeds of most legumes including pea [11,12,16]. In contrast, the seed coat or testa develops from maternal tissue surrounding the ovule and thus, functions at the interface between the mother plant and the embryo. Although fertilization events directly initiate embryo and endosperm formation, successful fertilization is also required to lift the repression of sporophytic genes encoding polycomb-type proteins, thereby inducing seed coat development [17]. Early embryo development is controlled by communications with surrounding maternal tissue, with signals from the maternal plant transmitted through the seed coat and endosperm [18,19]. The interdependence of developmental programs amongst seed sub-compartments is further highlighted by the rapid degeneration of the early seed coat in the absence of successful fertilization [17].

Interdependencies between early-stage embryo, endosperm, and seed coat persist, as the seed develops, facilitating coordinated metabolism, resource allocation, and the differentiation of the highly specialized tissues within seeds. Seed development in pea and other legumes is commonly subdivided into a pre-maturation/pre-storage/cell division phase, a transitional phase, a maturation/storage phase, and desiccation phases [20]. These phases distinguish key switches in embryonic developmental processes, which are governed by changes in the expression of genes and regulatory programs, hormone, and sugar signals and are inherently linked to processes occurring in the seed coat [20]. Among these transitions, the shift in embryonic growth from cell division to cell expansion and away from maternal regulation towards filial independence distinguishes the early phases of seed maturation in which the seed coat plays a major role. In later phases of maturation, seed development is characterized by the accumulation of nutrient stores, particularly starch and proteins, within embryonic cotyledons, supported by an influx of sucrose and followed by desiccation and dormancy. In pea, the genetic regulation of seed maturation was examined by profiling the transcriptional dynamics of seed development in two pea lines with contrasting maturation rates [21]. This study successfully identified transcriptional markers for early maturation and highlights the need for continued genetic resource creation in pea to progress understandings of the mechanisms regulating seed differentiation. Here, we discussed the key role played by the seed coat In *P. sativum* seed development, with a focus on advances made in the genetic and molecular characterization of pea and other legumes and the potential of this key seed tissue for targeted improvement and crop optimization.

## 3. Sensing and Transmission through the Seed Coat

Fortifying seeds with adequate resources to support independent life and ensure successful reproduction requires significant input from the parent plant. Nutrients mobilized from source organs of the parent plant must move from the maternal tissues of the ovule, through the funiculus connecting with the seed coat and into the embryo. Further, feedback provided by the developing embryo and endosperm requires transmission to the parent plant. This requirement for the two-way flow of materials and signals between the parent and the developing embryonic offspring is predominantly moderated and facilitated by the seed coat. The seed coat plays a critical multi-functional role as a conduit for communication between plant generations, supporting embryo formation and fortification. Thus, efforts to target seed traits of agronomic importance should recognize the potential of the seed coat as an avenue for modifying seed quality and its productivity [22].

## 4. Maternal Inputs to the Developing Pea Seed

In pea and many legumes, long-distance phloem transport from source organs culminates in the seed, with nutrient stores particularly enriched in the embryonic cotyledons. In most legume species, including pea, seeds are borne within pods (also called legumes), requiring seed-destined nutrients to pass through this sheath [19]. Enclosure of pea seeds within a pod creates a microenvironment with relatively high humidity and elevated CO_2_ concentrations over atmospheric levels [23], which greatly impacts the conservation of carbon released by seed respiration and reduces transpiration rates. This has important implications for the transport of photoassimilates from the parent plant to developing seeds, as reduced transpiration requires the import of most minerals via the phloem, rather than xylem [24,25]. Beyond nutrient shuttling, pod functions are numerous and often complementary to seed coat functions, serving as a protective barrier to developing embryos, modulating seed dispersal and supplementing photosynthetic outputs [26]. Altogether, the pod envelope contributes significantly to seed development in pea and must be considered alongside efforts targeting seed coat trait improvement.

As the link between the parent and the offspring, the seed coat plays a critical role in coordinating nutrient flow into the seed. In pea, the flux of nutrients and water from the maternal parent funiculus occurs through the chalazal region, supported by a concentric amphicribral vascular bundle of xylem surrounded by phloem [18]. The simple vasculature of the chalazal vein diverges into two phloem-based branches that supply the pea seed coat [27]. The maternal funiculus provides a conduit for resources entering the chalazal region but is absent from the mature legume seed, detaching during late stages of maturation and drying to leave the hilum scar. In legumes, the bulk of nutrient assimilates including amino acids, sugars, and micronutrients, as well as certain phytohormones, are phloem-imported. Importantly, this vascular system terminates in the chalazal region of the seed coat, and thus, after phloem unloading in pea seed coat parenchyma (Figure 1C), assimilates destined for the embryo must traverse the apoplastic space [28]. In pea, the intercellular spaces of the apoplast between the maternal and filial sub-compartments of the seed are predominantly aqueous in nature, containing high solute concentrations conducive to nutrient diffusion towards cotyledons [18,29,30]. As seed development progresses and the control of development shifts from maternal to filial-based, the mechanisms supporting nutrient transfer from the mother plant to the embryo are programmed accordingly [19].

Early-phase embryonic growth is strongly coordinated by maternal controls. The seed coat impacts embryo growth and ultimate seed size through physical and enzymatic controls, suppressing its own growth and mediating the flow of nutrients to the embryo through enzymatic conversions and transient storage of sieve-imported assimilates [19,20,31]. In *Medicago truncatula*, a number of proteases that accumulate preferentially in the seed coat have been hypothesized to mediate protein turnover and nitrogen remobilization, supplying developing embryos with the amino acid building blocks for protein synthesis [32]. Sensing and partitioning of carbon in young legume seeds is largely mediated by seed coat-borne invertases. Intriguingly, seed coat-borne soluble/vacuolar and cell wall-localized invertases appear to provide unique but complementary functions in mediating embryonic growth, as stress sensors capable of adjusting seed production rates based on resource availability and in altering the sucrose-to-hexose ratio in the seed apoplasm, respectively [33,34]. In the case of cell wall invertases that metabolize phloem-imported sucrose into glucose and fructose, invertase-generated monosaccharides represent key players in modulating early embryonic development and sink strength, with the import of these hexoses into the embryo shown to promote embryonic cell division. Further, high hexose ratios have been associated with the induction of specialized transfer cell differentiation in the embryonic epidermis of *Vicia faba* to support embryo nutrient uptake [35,36]. Cell wall inhibitor proteins have been identified as regulators of cell wall invertase activities in the seed coat of pea. Interesting discoveries associated to the duration of cell wall invertase activities with seed size in *Vicia faba* suggest that in legumes, targeting seed coat-localized invertases, either directly or through the modulation of their regulators, could provide a mechanism for modulating cell division in early stages of embryo development, ultimately impacting embryo size and the potential for modulating seed biofortification [33]. Thus, seed coat cell wall invertases and their regulators mediate assimilation unloading and carbon partitioning in the early legume seed.

Whereas maternal inputs predominantly regulate early embryo growth, transition into the maturation phase involves a switch to the embryonic control of development. This includes the cellular differentiation of embryonic tissue, enabled by a shift away from the import of hexose sugars supplied by seed coat cell wall invertases, towards sucrose import [20,34,37,38,39]. In legumes, specialized filial transfer cells that support nutrient uptake from the apoplast differentiate as early as the transition phase. In pea, transfer cells line the epidermal surfaces of cotyledons in association with zones of seed coat nutrient unloading, and feature extensive wall ingrowths for surface area expansion and a plasma membrane enriched with assimilated transport-proteins [40,41,42]. Specialized transfer cells along with the seed coat also differentiate to support efflux to the apoplast and nutrient flux across the maternal−filial interface; however, the extent of their specialized differentiation varies amongst legumes [43]. Pea seed coat parenchyma (Figure 1C) lining the apoplast appears branched with large intracellular spaces and may support efflux in the vicinity of the embryo [18,40,42]. The differentiation of embryonic epidermal cells into transfer cells is accompanied by substantial sucrose uptake and a shift in embryonic sink strength, ultimately shifting embryonic development into a phase of maturation marked by cell elongation and storage [20]. The development of sucrose uptake mechanisms supported by transfer cell differentiation facilitates a shift away from the embryonic cell division phase of embryonic growth, under invertase-generated high hexose conditions, towards an elevated sucrose state in the embryo, enabling growth driven by cell expansion and triggering maturation of the embryo. Evidence of the importance of this epidermal differentiation for pea embryonic development has been demonstrated by the E2748 mutation of an unknown gene, with highly vacuolated mutant cotyledon epidermal cells that form tight associations with neighboring tissues and lack characteristic transfer cell morphologies. The absence of transfer cells in E2748 pea mutants inhibits uptake by cotyledons, as evidenced by reduced symplastic tracer mobility, which together with severe callus-like embryonic growth defects supports a critical role for transfer cells in sucrose uptake and pea embryo development [41]. The gene perturbed by the E2748 mutation remains unknown, highlighting the vast knowledge gaps that remain surrounding mechanisms of flux in legume seed development. Identifying the genetic regulation of nutrient partitioning mechanisms within the pea seed and the role played by the seed coat presents opportunities for modulating flux in favor of nutrient stockpiling within seeds, to adjust the profile of seed content for targeted biofortification and ensure efficient use of inputs, such as fertilizers.

Targeting source-to-sink translocation for improved carbon and nitrogen assimilation and partitioning has been touted as a leading strategy to significantly impact crop yields, with demonstrated potential in several species [44]. The seed coat’s function as a portal between generations situates this tissue as a critical target amongst efforts to enhance nutrient assimilation and partitioning in seeds. Recent genome-wide identification of sugar transporters in the Fabaceae family has provided an extensive resource to guide transporter identification in pea, highlighting numerous monosaccharide transporters (MSTs), sucrose transporters (SUTs), and sugar will eventually be exported transporters (SWEETs), with putative but largely unknown functions in coordinating carbon allocation and yield in pea [45]. In pea, several studies targeting the source-to-sink flux as a mechanism for improving plant resource allocation and pea yield have produced promising improvements by overexpressing selected transporters [46]. In particular, studies employing the “Push-and-Pull” approach to facilitate phloem loading in source tissues and unloading at the sink have positively impacted pea seed yields [46,47,48]. The dual overexpression of the SUT1 sucrose transporter in the leaf phloem and embryo transfer cells of pea enhances resource partitioning in the seed, raising sucrose and protein levels in developing seeds [46]. Producing pea seeds rich in protein requires efficient import of nitrogen fixed and assimilated in the roots and remobilized from the leaf and other vegetative source organs [49]. In pea, nitrogen stored in source organs, such as leaves, moves through the phloem primarily in the form of amino acids, with import into developing seeds putatively requiring the transport protein, AMINO ACID PERMEASE1 (AAP1). Targeting the potential bottlenecks in the flow of amino acids in pea, the dual overexpression of the *AAP1* in the leaf phloem and into the embryo where transport-facilitated uptake occurs improves seed yields and protein content [47]. Importantly, *AAP1*-overexpressing plants outperform their wild-type counterparts under abundant and limiting N treatments, with improvements in nitrogen use efficiency to complement increased pea seed biomass and elevated storage protein content [48]. Thus, determining the function of many nutrient transporters known in pea and shifting their expression at key flux junctions, such as the seed coat, offer promising avenues for growing high-yielding, nutrient dense peas with decreased synthetic inputs and detrimental environmental impacts. Targeting transporters in the seed coat to modulate nutrient import, modification, and transient storage similarly poses great potential for crop improvement [44].

## 5. The Multi-Layered Legume Seed Coat

The architecture of the seed coat supports its multi-functional and dynamic role in seed development, as a conduit for nutrients and signals, as a site of synthesis, modification, and accumulation of metabolites, and as a protective capsule regulating dormancy and longevity. The elaborated differentiation of legume seed coat cells into a multi-layered tissue (Figure 1C) serves a plethora of functionalities in support of seed formation, which can impact the final food product characteristics including nutritional content, flavor, shape, and texture [46]. Many of the genetic and molecular mechanisms governing seed coat differentiation have been well established in model plant species such as *Arabidopsis thaliana* and the legume model *Medicago trunculata* [50]. Global gene expression analyses in *Glycine max* L. and Medicago have identified numerous seed coat-specific genes and suggest that the genetic inputs governing legume seed coat development surpass even embryonic transcriptional complexity [51,52]. This differentiation of the seed coat includes sequential cell death in each of the layers of legume seed coats, as demonstrated in soybean [51]. Even in death, the seed coat supports embryo formation, as cells of the outer integument seal and structurally protect the seed, while the crushing of inner integument parenchyma cells and programmed cell death of seed coat transfer cells sustain embryonic growth through nutrient transfer and the release of spatial restraints [19,53]. In maize, programmed cell death in the seed coat chalazal region occurs early in seed formation, coordinates with endosperm cellularization and supports intergenerational transfer mechanisms [54]. Genomic resources for model legumes such as *Medicago truncatula*, together with pea-specific data, promise continued growth for the identification and characterization of seed coat target genes and their functionalities and impact on seed traits in pea [51,52,55].

Fertilization triggers the differentiation of the ovule epidermis into the ovular integuments that together with the chalazal tissue differentiate into the seed coat [56]. The development of the seed coat and endosperm is initiated before embryonic programs in legumes [20]. Legumes such as pea have bitegmic ovules with an inner and outer integument, and as seed development progresses, each integument features a sub-specialization that supports the diverse functionalities of the seed coat [18,19,31]. In pea, the inner integument comprises layers of chlorenchyma, ground, and branched parenchyma cells, which serve a transitory nutritive role [19]. Although crushed at maturity, the inner integument hosts important maternal processes that impact embryonic development and final seed size [18,57,58]. The analysis of an ADP-Glc pyrophosphorylase mutation in pea revealed the importance of transient starch storage in the inner layers of the seed coat for promoting sink strength in support of embryonic growth [59]. In addition, the outer integument of the *P. sativum* seed coat is capable of starch granule accumulation during mid-stage embryogenesis [60]. The innermost layer of the seed coat, known as the boundary layer, comprises specialized branched parenchyma or transfer cells, critically positioned between maternal and filial tissues as discussed above. The location of the boundary layer supports the expression of cell wall invertases and transporters in these cells.

In contrast to the collapse of the inner integument parenchyma in developing legume seeds, the outer integument forms a structurally impressive and impermeable boundary around the seed (Figure 1C). The outer integument contains two prominent cell layers, an epidermis of macrosclereids (also known as Malpighian or thick-walled palisade cells) and sub-epidermis or hypoepidermis of osteosclereids (also known as pillar or hourglass cells) (Figure 1C) [19]. In pea, the elongated macrosclereid layer features terminal caps composed of a thick cuticle and modified cell wall, providing this hard-seeded species with an impermeable barrier across the seed surface [61]. Uneven secondary cell wall thickenings are also featured in the subepidermis, which create bone-shaped osteosclereids separated by air-filled pockets between the epidermal and inner (crushed) parenchyma that are conducive to desiccation and gas exchange [19]. Beneath the seed coat, the closely associated outer layer of the endosperm, known as the aleurone, additionally supports legume seed development.

## 6. Seed Dormancy, Imbibition, and Permeability

The non-permeable nature of stone or hard seeds is a trait determined by the seed coat and is a common characteristic in the Fabaceae family, including the Pisum genus [62,63,64]. The water-impermeable nature of the seed coat mediates imbibition and thus, provides wild legume seeds with a mechanism to remain physically dormant until conditions favor successful germination [65]. Therefore, the permeability of the seed coat represents an important trait for breeders interested in selecting legume cultivars with rapid, uniform, and predictable imbibition and dormancy release to encourage standardized germination and growth [19]. Despite the significant loss of hardseededness in domesticated legumes, the mechanisms underlying physical dormancy and dormancy-breaking cues in legumes are poorly understood [66].

Seed permeability is a complex trait, governed by several quantitative trait loci and altered through external cues. Several anatomic features of the pea seed coat have been identified as contributors to water impermeability, including testa thickness and architecture, presence of compounds such as polyphenolics, and unique fatty acids in the seed coat and overlying cuticle [14,51,62,67]. In particular, the macrosclereid layer of the seed coat (Figure 1C) and the degree to which its outermost cuticle remains intact or cracks have been suggested as key determinants of seed permeability, including in hard-seeded legumes. However, the sites where water breaches the seed coat remain debated, and contradictory findings have been presented in pea [19,66,68,69]. A seed coat permeation study identified diffusion and bulk inflow as the prominent mechanisms for organic solute uptake operating in pea seeds during imbibition [70]. Studies in soybean, comparing genes expressed in permeable and impermeable cultivars, as well as genomic variants in cultivated and wild progenitor soybean species, have identified candidate targets and structural heterogeneities as potential targets of seed permeability or hardness traits in soybean [51,71]. Similarly, a thorough study of pea seed coat permeability provided insight into seed dormancy regulation by analyzing the path for water entry into non-dormant and dormant pea accessions [69]. In dormant pea seeds, a key role for the terminal cap of the seed coat macrosclereid cells (Figure 1C) in regulating water access was identified, demonstrating roles for the waxy subcuticular layer and light line elements in preventing water’s permeation into the seed, and thus may play crucial roles in regulating seed dormancy [69]. Conversely, analysis of anatomical features of the pea seed coat such as the micropyle, hilum, and strophiole, identified the strophiole as the major contributor to water permeation through the seed coat of non-dormant pea varieties [69].

**Figure 2:**
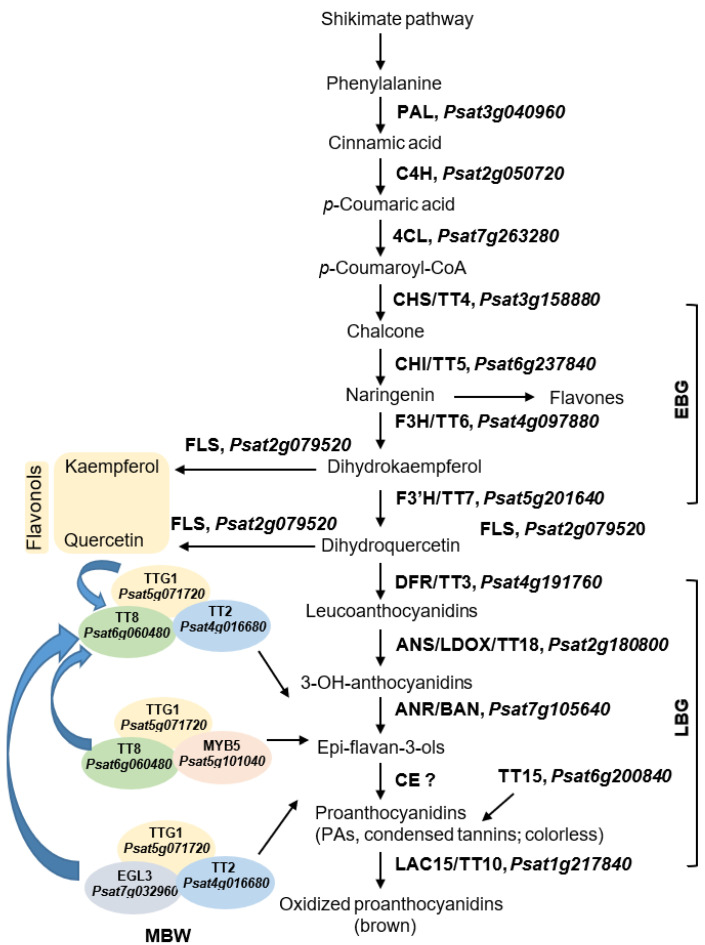
Scheme of putative tannin biosynthesis pathway in *Pisum sativum* L. seed coat. Flavonoid pathway was adapted from several references [73,74,75]. The gene ID was obtained using blastp against the pea reference genome sequence database (*Pisum sativum* L. version 1a) (https://urgi.versailles.inra.fr/Species/Pisum/Pea-Genome-project) (accessed on 15 June 2022). The threshold of 1 × 10^−7^ was used, and only the top hit gene with the best score is shown here. PAL, phenylalanine ammonia-lyase; C4H, cinnamic acid 4-hydroxylase; 4CL, 4-coumarate-CoA ligase; CHS/TT4, chalcone synthase; CHI/TT5, chalcone isomerase; F3H/TT6, flavonol 3-hydroxylase; F3′H/TT7, flavonol 3′-hydroxylase; FLS, flavonol synthase; DFR/TT3, dihydroflavonol reductase; ANS/LDOX/TT18, leucoanthocyanidin dioxygenase; ANR/BAN, anthocyanidin reductase; CE, condensing enzyme; LAC15/TT10, laccase 15; PAs, proanthocyanidins; TT2/8, transparent testa 2,8; TTG1, transparent testa glabra 1; EGL3, enhancer of glabra 3; EBG, early biosynthetic gene; LBG, late biosynthetic gene; MBW, MYB−bHLH−TTG1 complexes to regulate the expression of the LBGs.

In addition to anatomic discoveries associated with hardseededness, studies on the biochemical and genomic features of the seed coat have expanded the understanding of seed permeability and dormancy in legumes. Elevated phenolics and catechol oxidase activity in the seeds of wild peas (*Pisum elatius*) are correlated with reduced permeability over cultivated soft-seeded pea varieties [56]. Similarly, in Arabidopsis, the identification of seed coat genes encoding proteins functioning in coat color or proanthocyanidin biosynthesis (Figure 2) support a role for selected flavonoids in regulating seed permeability [26]. In *Medicago truncatula*, the role of a homeobox, KNOX4, in establishing hard seeds and physical dormancy was revealed through mutant analysis [72]. For more discussion on hardseedness and the impact of the seed coat on physical dormancy, including for legume species, the reader is referred to reviews [19,56,66,69]. Although much work remains to uncover the mechanisms that regulate seed permeability, hardseededness, and dormancy release in pea and other legumes, it is clear that the seed coat’s role in governing these traits is vital.

The degree of hardseededness conveyed by the seed coat (or hull) impacts the need for pea seed treatments, including soaking, cooking, and to a lesser extent milling, splitting, and removal of the seed coat by de-hulling [66,76,77]. Prior to human consumption, whole dried peas are soaked to promote seed coat and cotyledon expansion and cooked to alter structural and compositional features of the seed, including within the seed coat [76,78]. The physicochemical changes caused by extended cooking can have detrimental effects on the nutritious content of peas, including denaturing proteins, but improves pea quality and digestibility by reducing anti-nutritional constituents such as oligosaccharides linked to flatulence [6,76,79]. Phytate, a storage form of phosphorous in plants that is predominantly found in embryonic cotyledons and to a lesser extent in the seed coat in pea, is believed to chelate with minerals and proteins, impairing nutrient absorption [80]. However, chelation of phytic acid with cations such as calcium and magnesium is hypothesized to alter pectin composition and can therefore improve the breakdown of cell walls during cooking [76]. Future efforts to integrate desired traits from wild pea varieties into cultivated crops must consider the frequency of hardseededness conveyed by wild seed coats and the inheritance of this maternal trait, to ensure the inheritance of the desired seed coat trait in successive generations [81]. To satisfy consumer preferences and increase utilization of peas, future breeding and targeted improvement efforts must consider the unique contribution made by the thick seed coat and how the seed coat’s structural and compositional properties impact processing of the pea seed.

## 7. Contributions of the Seed Coat to Diet and Nutrition

The enriched nutrition provided by pea and many legume seeds is supported by the thick seed coat and well-developed embryo, key anatomic features that differ from the less developed embryo and abundant endosperm that characterize cereal species seeds [77]. Nutritious constituents of the pea seed, including protein and starch, predominately accumulate in filial tissues, while the seed coat provides soluble and insoluble fiber to the animal diet [82,83,84]. The legume embryo stores nutrient resources to support its growth and germination. These stored reserves predominantly accumulate in embryonic leaves or cotyledons, particularly during the late embryonic stages and the maturation phase of seed development, placing legume seeds among the top plant sources for nutritional value. In peas, embryonic nutrient stores are particularly rich in protein (estimated at 23–25%) and slowly digestible starch (approximately 50%). The relatively high levels of lysine, leucine, and arginine in legume seeds offer nitrogen (N)-containing essential amino acids that compliment cereal-based diet deficiencies [2]. This nitrogen fortification of legume seeds, although beneficial to human and animal dietary needs, will sustain seedling growth through the heterotrophic conditions of germination in advance of root−nodule associations with rhizobia bacteria to supply fixed nitrogen. The seed coat’s impact on the dietary value of pea seeds is complex, not only altering the bioavailability and digestibility of nutrients, but also contributing to benefits such as dietary fiber and bioactive phytochemicals. Cellulose and water-insoluble polysaccharides within the seed coat, as well as embryonic cell walls, provide significant dietary fiber and decrease pea starch digestibility [6,85,86].

## 8. Seed Coat Chemistry

Emerging data on the metabolite profiles of pea and the health benefits they confer are expanding the known nutritional value of these and other legumes [87]. The pea seed coat functions as a transient storage organ, retaining reserves of sugars and producing amino acids that are then transported into the developing seed [18,58]. In addition, pea seed coats house substantial metabolic diversity, serving as a production and storage site for a range of vitamins, minerals, and phytochemicals including phenolics, polyphenols, and terpenoids, as well as saponins, oxalates, and phytate [6]. Several studies have examined the specialized metabolites in pea seeds, including investigations of varietal variation, tissue-specific chemical profiles, and antioxidant activities [87,88,89,90]. For plants, these compounds serve an array of functions, defending against microbes, UV damage, and reactive oxygen species, as signaling molecules and by structurally fortifying maternal integument tissues. In pea, many of these compounds were formerly considered anti-nutritive, due to their impacts on pea protein digestibility and mineral bioavailability [91]. However, legume seed coats are gaining recognition for the range of health benefits offered to the consumer, due to the presence of natural antioxidants with anti-inflammatory, anti-carcinogenic, cardioprotective, and other beneficial activities that augment the protein-rich sustenance provided by their seeds [91]. Despite a growing appreciation for the nutritive value of peas and other legumes, molecular and genetic understandings of the mechanisms that support specialized metabolism within the major seed tissues and amongst cultivars are limited.

The pea seed coat provides a rich source of bioactive phenolic compounds, particularly in dark-colored seed varieties (Figure 1B), in which flavonoid compounds accumulate in abundance [84,88,89,90,92]. Flavonoid compounds are derived from phenylpropanoid metabolism and can be grouped into several subclasses, including anthocyanins, proanthocyanidins, isoflavonoids, and flavonols. Proanthocyanidins (PAs) or condensed tannins are polyphenolic flavonoids that accumulate in the seed coat of colored pea varieties (Figure 1A,B and Figure 2), predominantly in association with the cell wall of mature seeds. The biosynthesis of flavonoids in plants has been studied extensively (Figure 2), aided by the clear visual phenotypes produced by mutations perturbing anthocyanin or PA production [73]. In seeds, this mutant phenotype commonly alters seed coat pigmentation (Figure 1A,B), producing transparent testa. The physical separation of seed tissues can be achieved with relative ease due to large seeds and thick coats produced by pea, further facilitating studies on the diversity of flavonoids and other metabolites specific to pea seed coats (Figure 2) [93,94].

Pea varieties with different seed colors (Figure 1A,B) exhibit a range of differences in their detected phenolic compounds, including a number of flavanols in colored pea varieties (Figure 1A,B) [91]. In short, metabolite profiling studies that distinguish the pea seed coat from the embryo report enrichment of flavones and flavonols, PAs/tannins (Figure 2), and simple phenolics in the seed coat, in contrast to elevated catechin in pea cotyledons [6,90]. Comparisons between white and colored (yellow) pea varieties detect condensed tannins (Figure 2) present uniquely in colored seed coats, with higher levels of free, esterified and glycosylated phenolic acids and anthocyanins in colored seed coats, and different dominant phenolic acids in white vs. colored seed coats [6,88,89]. Although many of these metabolites accumulate transiently or are cell-wall-bound, the seed coat also maintains soluble chemical reserves that protect and nourish the growing embryo. In many legumes, the presence of PAs contributes to the hardseeded nature of the seed coat, and their oxidation in maturing seeds produces brown pigmentation that strengthens seed impermeability.

Flavonoid biosynthesis pathway genes have been found to affect the dormancy of Arabidopsis seeds [95], but less is known about such a role in legumes. Several genes encoding biosynthetic enzymes, transporters, or regulatory proteins required for PA accumulation in the seed coat have been functionally characterized in legume model species, although much work remains to be performed in pea. In *Medicago truncatula*, genes encoding PA biosynthetic enzymes and putative regulators have been identified [96], and a multi-drug and toxic compound extrusion transporter, called MATE1, functions as a flavonoid/proton antiporter [97]. The elevated expression of such genes within the Medicago seed coat supports a role in mediating PA biosynthesis within the coat [98]. Many gaps in our knowledge of PA formation in seeds remain, for example the subcellular sites of PA biosynthesis and precursor accumulation, the mechanisms of PA precursor condensation, and subcellular trafficking.

Efforts to modify the levels of beneficial bioactive components within pea seeds and their coats must consider bioavailability and the complex interaction of specialized metabolites with proteins, minerals, and other basic nutritional components. For example, efforts to fortify the seed coat with PAs can have undesirable outcomes, leading to a reduction in the minerals available to the diet due to cross-linking. Despite the health benefits associated with PAs, strictly targeting increased production and accumulation of PAs in peas can yield reductions in palatability and digestion of proteins and minerals. Reduced PAs similarly reduce health benefits, offering no relief of pasture bloat or improved animal growth. Thus, achieving pea varieties that balance PA content with protein and mineral composition is necessary to support optimal antioxidant benefits. In addition, the metabolic specialization observed amongst the three major tissues of pea seeds means modifying mechanisms that sequester compounds between seed tissues and/or alter sink-to-source relationships offer approaches to fine-tuning the physiological balance of a seed’s valued constituents. Understanding how the heterogeneous landscape of chemical resources is spatially distributed in pea seeds and the genetic programs that enable differential accumulation is increasingly possible and will open novel opportunities for targeted improvements.

## 9. Concluding Remarks

Climate changes threaten global food security, placing increasing pressure on the development of sustainable agricultural solutions to secure future food production and food security. With the forecasted increases in global temperatures and frequency of extreme events, data on the effects of abiotic and biotic stress on seed physiology, development, and metabolic output are needed to facilitate sustainable development and management of plant-based resources such as legume crops. As the consumption of seeds and seed products continues to rise, it is vital that we improve our understanding of resource allocation in legume crop species, to ensure quality improvements are targeted and effective and yield sustainable production and quality improvements. As the sheath that encapsulates the developing embryo, the seed coat’s roles are multifaceted and critical, sensing external cues, producing and transporting metabolites to nourish and defend the next generation and establishing dormancy and facilitating germination. The improvement of meal quality in pea is a complex challenge, requiring several complementary approaches aimed at increasing protein content, decreasing the anti-nutrition components (such as trypsin inhibitor, tannins, and phytic acid) and optimizing the content of nutritional components, i.e., prebiotic carbohydrates, dietary fiber, minerals, vitamins, and specialized metabolites. Thus, future pea seed coat research would benefit from an expanded understanding of the following: (1) regulatory gene networks controlling seed coat development and nutrition and anti-nutrition synthesis pathways; and (2) the crucial genes controlling seed coat traits and pea meal quality, including seed size, coat thickness and color, nutritional and anti-nutritional content, seed dormancy, imbibition, permeability, and germination.

## Data Availability

Not applicable.
